# Affinity-based enrichment strategies to assay methyl-CpG binding activity and DNA methylation in early *Xenopus *embryos

**DOI:** 10.1186/1756-0500-4-300

**Published:** 2011-08-18

**Authors:** Ozren Bogdanović, Gert Jan C Veenstra

**Affiliations:** 1 Department of Molecular Biology, Nijmegen Centre for Molecular Life Sciences, Radboud University Nijmegen, Faculty of Science, (Geert Grooteplein 28), Nijmegen, (6525 GA), The Netherlands

## Abstract

**Background:**

DNA methylation is a widespread epigenetic modification in vertebrate genomes. Genomic sites of DNA methylation can be bound by methyl-CpG-binding domain proteins (MBDs) and specific zinc finger proteins, which can recruit co-repressor complexes to silence transcription on targeted loci. The binding to methylated DNA may be regulated by post-translational MBD modifications.

**Findings:**

A methylated DNA affinity precipitation method was implemented to assay binding of proteins to methylated DNA. Endogenous MeCP2 and MBD3 were precipitated from *Xenopus *oocyte extracts and conditions for methylation-specific binding were optimized. For a reverse experiment, DNA methylation in early *Xenopus *embryos was assessed by MBD affinity capture.

**Conclusions:**

A methylated DNA affinity resin can be applied to probe for MBD activity in extracts. This assay has a broad application potential as it can be coupled to downstream procedures such as western blotting, fluorimetric HDAC assays and quantitative mass spectrometry. Methylated DNA affinity capture by methyl-CpG binding proteins produces fractions highly enriched for methylated DNA, suitable for coupling to next generation sequencing technologies. The two enrichment strategies allow probing of methyl-CpG protein interactions in early vertebrate oocytes and embryos.

## Background

DNA methylation is an epigenetic modification mostly associated with transcriptional repression in vertebrate genomes. In vertebrates, methylation events occur predominantly within the context of CpG dinucleotides, which in mammals by and large are methylated [1,2]. The repressive signal inferred by DNA methylation is further interpreted by methyl-CpG binding domain (MBD) proteins that bind sites of DNA methylation and can recruit complexes with histone deacetylase (HDAC) activity to silence transcription [3-6]. The MBD was initially described as the minimal part of the MeCP2 protein required for methylated DNA binding [7]. Four remaining family members (MBD1, MBD2, MBD3 and MBD4) have been discovered on the basis of homology searches with the MeCP2 MBD amino acid sequence [8]. In addition, several structurally unrelated methyl-CpG binding proteins have been identified [9,10]. Mutation or knockdown of DNA methyltransferases and MBD genes result in severe developmental phenotypes [11-14]. Mutations in the MBD family founder, MeCP2, are the cause of Rett Syndrome (RTT), a grave neurodevelopmental disorder which due to its X chromosome linkage almost exclusively affects females [15,16]. In post-mitotic neurons, the brain-derived neurotrophic factor (BDNF) promoter III is controlled by phosphorylation-dependent MeCP2 binding [17,18]. Membrane depolarization results in MeCP2 phosphorylation, carried out by the CamKI kinase, which diminishes its binding affinity for methylated DNA [19,20]. A number of methods to study DNA-protein interactions have been developed over the years. For example, chromatin immunoprecipitation (ChIP) coupled to microarray hybridization or Next-Generation Sequencing technologies has facilitated the generation of genomic maps of transcription factor binding sites [21,22]. Other widely used methods to study DNA-protein interactions involve DNA footprinting, EMSA (Electrophoretic Mobility Shift Assay), southwestern blotting (SW), DNA affinity chromatography and DNA-protein crosslinking *in vitro *(DPC) [23,24] DNA affinity precipitation is an efficient method for the analysis and purification of proteins displaying sequence specificity for DNA [25,26]. Such assays can be used to study binding affinity and recruitment specificity of either recombinant or cellular proteins [27,28]. Recently, important progress has been made in understanding the interactions of modified histone tails with transcriptional regulators [29-31]. These studies have combined peptide affinity pull-downs and quantitative mass spectrometry enabled by SILAC (Stable Isotope Labeling by Amino Acids in Cell Culture) [32]. It will be of great importance to define proteins recruited to methylated DNA during early development. For over a century amphibians from the genus *Xenopus *have been the organisms of choice to study developmental processes. The main reason for this is external embryonic development as well as the relatively large size of the embryos themselves which allows easy experimental manipulation. A number of studies involving DNA methylation and methyl-CpG binding proteins established *Xenopus laevis *as an important model for epigenetics studies [9,33-35]. Sequencing of the *Xenopus tropicalis *genome rendered this organism suitable for (epi)genomics studies of the early vertebrate development [36-38]. Here we describe an efficient method for methylated DNA affinity precipitation of MBDs from *Xenopus *oocytes. Using *in vitro *methylated DNA oligonucleotides bound to magnetic beads and conditions that promote methylation-specific binding, MeCP2 and MBD3 were pulled down. These methylated DNA affinity precipitation assays have a broad application potential as they can be combined with a number of biochemical techniques and used in different model systems. Furthermore, to test the ability of MeCP2 to affinity-precipitate methylated DNA from early *Xenopus *embryos, the immobilized MBD domain of MeCP2 can be used to enrich for methylated genomic DNA [39-41]. The fractions enriched for methylated DNA, due to their high recovery rate, are suitable for coupling to next generation sequencing technologies.

## Methods

### Oocyte extract preparation

*Xenopus laevis *oocytes were collected in an eppendorf tube and homogenized in ice cold lysis buffer (20 mM Tris (pH = 8,0), 70 mM KCl, 1 mM EDTA, 10% glycerol, 5 mM DTT, 0,125% NP40, Roche Complete protease inhibitors). The extract was centrifuged for 5 minutes at 4°C at maximum speed. The supernatant was collected and quick-frozen in liquid nitrogen. The final oocyte concentration in the extract was 0.5 oocyte equivalents per μL assuming that one oocyte corresponds to 1 μL.

### MeCP2-MBD *in vitro *transcription and translation

The pT7TS-5'MBD construct, encoding the first 176 amino acids of *Xenopus laevis *MeCP2, was obtained by PCR amplification (primers 5'-gtatccgtcgacaattcggcacgagagaaaATG and 5'-acaagaggatcctcaGGCTTTCGGTTGCTTCTGTTCC), digestion of the PCR product with SalI and BamH1, blunting and ligation in the EcoRV site of pT7TS. *In vitro *transcription of the pT7TS-5'MBD was performed using the mMESSAGE mMACHINE T7 kit (Ambion). The *in vitro *translation was achieved using 1 μg of RNA in a total volume of 50 μL (Flexi Rabbit, Promega). The binding assay was performed in 150 μL ABCD buffer 20 mM Tris (pH = 8,0), 1 mM EDTA, 5 mM DTT, 5 mM MgCl_2_, 100 mM KCl, 0,125% NP40, protease inhibitors) with 6 μL of the *in vitro *translation reaction, using the protocol described below.

### DNA affinity precipitation

The DNA probe was obtained by annealing of 50 μg of two complementary oligonucleotides (5'-GATCCCGGAGTTAA and 5'-GATCTTAACTCCGG), modified from Klose et al. [42]. The annealed probe was phosphorylated with T4 polynucleotide kinase (New England Biolabs) for 2 h at 37°C, purified by phenol-chlorophorm and precipitated with ethanol. The concatamerization reaction was performed at RT for 4 h with T4 ligase (Promega) in the presence of 10 mM ATP to replenish the ATP from the ligase buffer. The ligation process was monitored using 1% agarose gel electrophoresis. The reaction was ethanol precipitated for one hour at -20°C. After DNA precipitation, two biotinylated nucleotides, biotin-16-dUTP and biotin-14-dATP were used to fill in the overhangs in a polymerization reaction using the Klenow fragment (New England Biolabs). All the nucleotides in the reaction were present at a concentration of 0.1 mM. The reaction was allowed to proceed for 45 minutes at room temperature. The biotinylated DNA was then purified using a gel extraction kit (Qiagen) and the samples were recovered in 30 μl elution buffer. The biotinylated oligos (~50 μg) were methylated with SssI methyltransferase in a 500 μl reaction for 2 h at 37°C. Phenol-chlorophorm extraction was used to clean the methylated DNA which was then precipitated by ethanol. The methylated probe was then coupled to streptavidin beads (DYNA beads M-280). For each 3 μg of DNA, 30 μl of the streptavidin beads was used. The beads were washed 3X with the WB (1 M NaCl, 20 mM Tris pH 8.0, 1 mM EDTA) buffer. After washing, 3 μg of the methylated probe (10 μl) was mixed with 40 μl of WB buffer. Out of the 50 μl of the WB buffer, 10 μl were separated for subsequent gel analysis while the remaining 40 μL was added to streptavidin beads. The mixture was incubated on a thermomixer set at 30°C for 30 minutes with shaking. Oocyte extracts (100 μL) were coupled to the immobilized probe in affinity precipitation (AP) buffer (20 mM Tris pH 8.0, 1 mM EDTA, 5 mM DTT, 5 mM MgCl_2_, 100 mM KCl, 0.125% NP40, protease inhibitors) in a 1:1 ratio and left rotating over night at 4°C. After incubation the mixtures were washed with washing buffer (20 mM Tris (pH = 8,0), 1 mM EDTA, 5 mM DTT, 5 mM MgCl_2_, 100-200 mM KCl, 0,125% NP40, protease inhibitors) for three times and the bound fraction was eluted with 50 μL 1X Laemmli sample buffer and analyzed by western blotting.

### Western blotting

The eluate (25 μl) was resolved on 10% polyacrylamide gels and subjected to western blotting. The α-MeCP2 antibody (V17-1) was raised against the C-terminal peptide MeCP2 peptide of *Xenopus laevis *(PRPTREEPVDTRTT). The final serum was affinity purified and used in a 1:1000 dilution. The ISWI, Mi-2 and MBD3 antibodies have been described before [5,43,44]. The western blotting protocol was performed as described before [45].

### Genomic DNA isolation and sonication

*Xenopus tropicalis *embryos (n = 50) were homogenized in 625 μL (12.5 μl/embryo) of homogenization buffer (20 mM Tris pH 8.0, 100 mM NaCl, 15 mM EDTA, 1% SDS, 0.5 mg/ml proteinase K). The homogenate was then incubated for 3 h at 55°C. The tubes were gently inverted after every hour of incubation. Two Phenol/Chlorophorm/Isoamylalcohol (25:24:1, PCI) extractions were performed using the same amount (625 μL) of PCI and inverting the tubes gently. The samples were spun for 5 minutes at 13000 rpm in a table-top centrifuge following every PCI extraction. The DNA was precipitated by adding 1/5 volume of 4 M NH_4_Ac and 3 volumes of cold 96% ethanol and left on ice for 1 h. The DNA precipitate was then centrifuged for 20 minutes at 4°C and 13,000 rpm in a table-top centrifuge. The pellet was washed with 500 μL of 70% ethanol and centrifuged for 5 minutes, 13,000 rpm at RT. The pellet was then resuspended in 200 μL of TE buffer with 1 μl of RNAse A (20 μg/μl) and left at RT for 30 minutes. The reaction was precipitated with 0.1 volumes of 4 M NH_4_Ac and 1 volume of isopropanol on ice for 1 h. The precipitate was centrifuged for 20 minutes at 4°C at maximum speed in a table-top centrifuge and washed with 70% ethanol. The pellet was resuspended in 100 μl of DNAse/RNAse free water. Two samples consisting of 50 embryo equivalents were pulled together to obtain a 200 μl sample with a concentration of ~ 0.1 μg/μL. The sample was sonicated for 15 minutes (13 seconds on, 16 seconds off, maximum output) in a Bioruptor (Diagenode) device.

### Methylated DNA affinity capture (MethylCap)

Genomic DNA was sheared by sonication to ~ 500 bp fragments. Methylated DNA was affinity purified using the H6-GST-MBD fusion protein (Diagenode) following the manufacturers' protocol with one modification. The DNA was eluted in several steps with increasing NaCl concentrations (150 μl): 2 × 200 mM, 1 × 300 mM, 1 × 500 mM, 1 × 600 mM and 1 × 700 mM. The recovery of methylated DNA from six genomic targets was calculated by quantitative PCR, whereas the concentration of DNA in each eluate was obtained by Picogreen measurement.

## Results

### Immobilized DNA preparation

**Figure 1:**
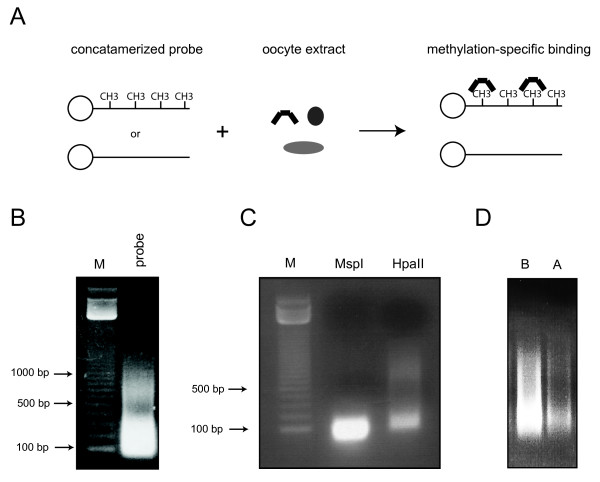
**Methylated DNA affinity precipitation**. **(A) **A methylated, concatamerized oligo is incubated with oocyte extract to probe for proteins with methyl-CpG binding activity. After performing the necessary washing steps, the eluate contains proteins specifically binding methylated DNA. **(B) **The concatamerization of the oligo is monitored by agarose gel electrophoresis. The majority of concatamerized DNA consists of fragments ranging in size from 100 - 300 bp. **(C) **The extent of oligo methylation is measured by restriction digestion. MspI enzyme cuts both methylated and unmethylated DNA whereas its isoschizomer HpaII cuts only unmethylated DNA. **(D) **The binding of the biotinylated oligo to streptavidin beads is measured by agarose electrophoresis. An aliquot of the probe before incubation with streptavidin beads (before - B) is compared to a probe aliquot after 30 minutes of incubation time (after - A).

To study the binding affinities of MBDs in *Xenopus*, small-scale DNA affinity precipitation was applied using the sequence GATC-CCGG-AGTTAA which features a single CpG dinucleotide [42]. A probe containing 11 CpG dinucleotides (6(GAC)-CCGG-6(GAC)) yielded similar results but proved more difficult to concatamerize, probably due to high CpG density of the fragment. The double-stranded oligonucleotide was methylated, concatamerized, immobilized and incubated with oocyte extracts (Figure 1A). After performing the washing steps, the proteins that bind methylated DNA were eluted. Performing the same experiment in parallel with an unmethylated oligonucleotide is a necessary control ensuring the binding occurs due to template DNA methylation and not sequence specificity alone. Following annealing and phosphorylation, the probes were concatamerized. Concatamerization is an essential step as DNA may be degraded by the exonucleases present in oocyte extracts. The concatamers ranged in size from 100 bp - 1000 bp with the majority falling between 100 - 300 bp (Figure 1B). To improve the concatamerization, a second round of phosphorylation and ligation may be performed. Concatamerized probes were then labeled with two biotinylated nucleotides (biotin-16-dUTP and biotin-14-dATP) to ensure the maximum binding efficiency in a Klenow fill-in reaction. Following biotinylation, the probes were *in vitro *methylated by the SssI enzyme. The extent of the methylation reaction was examined by MspI and HpaII digestion (Figure 1C). Such a digestion reaction should be performed to ensure that the probe is efficiently methylated and the concatamerization worked properly. High molar concentrations of oligos during the ligation reaction might induce concatamerization artifacts which then would not get efficiently cut by the MspI enzyme. Following the *in vitro *methylation reaction the probe was coupled to streptavidin beads. After incubation, 10 μl of the binding reaction was compared to 10 μl of the input by 1% agarose gel electrophoresis (Figure 1D). Immobilized DNA templates coupled to streptavidin beads can be kept at 4°C in the same buffer used in the washing and binding steps.

### Binding of the in *vitro *translated MBD domain to immobilized templates

**Figure 2:**
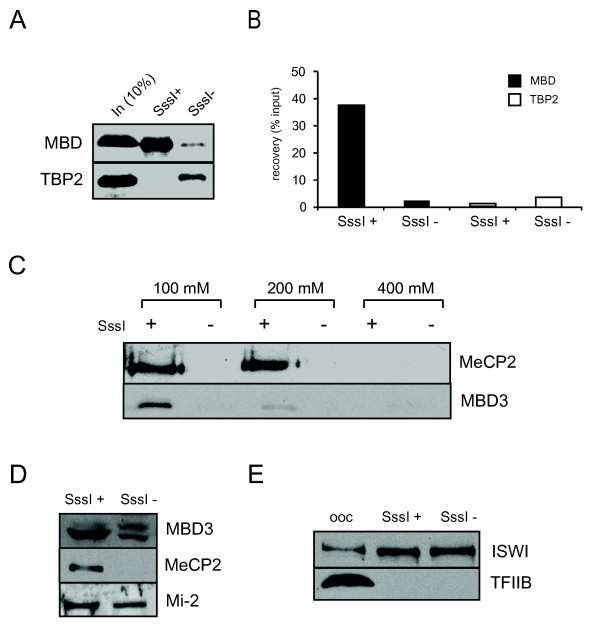
**DNA affinity precipitation of recombinant and endogenous oocyte extracts proteins**. **(A) ***In vitro *translated, S^35 ^labeled MBD domain of MeCP2 specifically binds the methylated immobilized probe (SssI+). Conversely, the negative control (TBP2) displays only residual affinity for non-methylated DNA (SssI-). **(B) **Quantification of DNA methylation-specific binding using the Image-quant software. ~35% of the recombinant MBD domain is recovered after washing and elution from methylated DNA oligos. **(C) **Western blotting of eluates obtained after 100 mM, 200 mM and 400 mM KCl washes. MeCP2 tolerates 100 mM and 200 mM KCl concentration in the washing buffer whereas most of the MBD3 is eluted with 200 mM salt. **(D) **DNA affinity precipitation of MBD3, MeCP2 and Mi-2 from oocyte extracts. MeCP2 displays strong preference for methylated DNA, whereas Mi-2 and MBD3 bind both methylated and unmethylated DNA. The short form of MBD3, however, displays a preference for methylated DNA. **(E) **Establishment of positive (ISWI) and negative (TFIIB) controls for DNA affinity precipitation.

To test whether the templates have assembled properly and whether the system is functional, radiolabelled (^35^S) MeCP2-MBD (containing amino acids 1-176 of MeCP2) and TBP2 (negative control) [46] were generated by *in vitro *translation using the rabbit reticulocyte lysate system. The binding occurred in the affinity precipitation buffer at 4°C. The reaction was assembled and left overnight on a rotating platform. The following day, 10% of the input and 50% of the eluate of the binding reaction were loaded on a 10% polyacrylamide gel (Figure 2A). After exposure the visible bands were quantified using the Image-quant software (Figure 2B). A high specificity for DNA methylation-specific binding was observed; ~ 40% of the MBD domain was recovered from the methylated probes while the recovery from the non-methylated control was below 3%. TBP2 displays very low affinity (< 1% recovery) for the methylated template whereas the binding to the unmethylated template was in the order of ~4%.

### DNA affinity precipitation of MBDs from oocyte extracts

To optimize washing and elution conditions, the protein-DNA complexes formed during an overnight incubation of oocyte extracts and immobilized probes, were washed with increasing concentrations (100/200/400 mM) of KCl and eluted with Laemmli buffer. Once eluted with Laemmli buffer, the samples were spun down to separate the beads from the supernatant and the eluate was loaded on SDS-PAGE gel. Western blot analysis revealed strong preference for methylated DNA binding of both MBD3 and MeCP2 (Figure 2C-D). The mammalian MBD3 is not able to bind methylated DNA due to an insertion in the MBD. *Xenopus *MBD3, however, is present in a short and a long form, the former of which is able to specifically bind methylated CpGs [5,47]. Whereas MeCP2 tolerates 200 mM KCl in the washing buffer, most of MBD3 is eluted at such conditions (Figure 2C). MeCP2 and the short form of MBD3 display a strong preference for methylated DNA (Figure 2D), in contrast to Mi-2 which can bind both methylated and unmethylated DNA. MBD3 and Mi-2 form part of the same complex [5]; the difference in binding may be explained by a variable composition of the Mi-2/NuRD complex [48-50] and methylation-independent binding of DNA by the Mi-2/NuRD subunit MTA1 [5]. Finally, a negative control for DNA methylation pull-downs was established. Any endogenous protein that does not bind directly to DNA might serve as a negative control in these assays. TFIIB is one of the general transcription factors involved in RNA Polymerase II pre-initiation complex formation [51]. Our assays identified it as a suitable negative control as it does not bind immobilized oligo templates independent of their methylation status (Figure 2E). In the same experiment we also tested the binding of ISWI, another evolutionarily conserved ATP-dependent chromatin binding protein and established its uniform binding to methylated and unmethylated DNA [44,52] Altogether, these results establish *Xenopus *oocytes as an efficient system to probe for interactions of methylated DNA and proteins with methyl-CpG binding activity. Furthermore, we provide a number of controls that can be used in such assays to control for unspecific protein-DNA interactions.

### Methylated DNA affinity capture (MethylCap)

**Figure 3:**
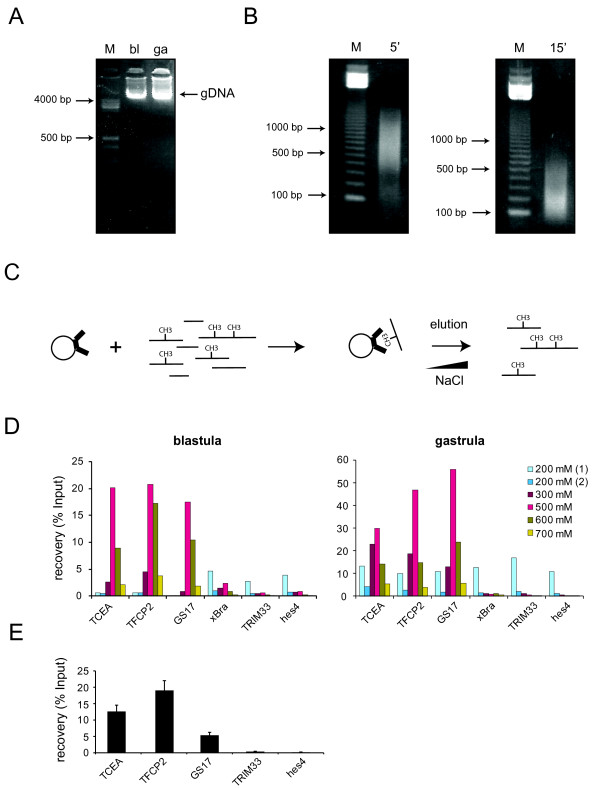
**Genomic DNA isolation and sonication**. **(A) ***Xenopus tropicalis *genomic DNA isolated from blastula (bl) and gastrula (ga) embryos. **(B) **Genomic DNA analyzed after 5 minutes or 15 minutes of sonication. Genomic DNA sonicated for 15 minutes yields fragments of 100 - 300 bp. **(C) **A schematic representation of methylated DNA affinity capture (MethylCap). Methylated DNA is bound by the immobilized methyl-CpG binding domain of MeCP2, washed and eluted with increasing NaCl concentrations. **(D) **PCR quantitation of methylated DNA recovery. DNA methylation targets (*tcea*, *tfcp2 *and *gs17*) mostly elute at high NaCl concentrations (500 mM, 600 mM and 700 mM) whereas the non-methylated targets (*Xbra*, *trim33*, *hes4*) are washed out with the first washing step (200 mM). **(E) **The methylation status of genomic targets *tcea*, *tfcp2*, *gs17*, *trim33 *and *hes4 *was validated by MeDIP (methylated DNA immunoprecipitation). The error bars represent standard deviation (SD) of two experiments.

A number of techniques to study DNA methylation on a genomic scale have been developed through the last decade. These include: Methylated DNA immunoprecipitation (MeDIP) [53-55], bisulfite sequencing [56,57] and methylated DNA affinity capture (MethylCap) [38,41,58]. These methods can be coupled to next generation sequencing technologies to identify the sites of genomic DNA methylation [40]. MethylCap is an affinity-based technique in which the immobilized methyl-CpG binding domain of one of the MBDs is used to capture fragments of methylated DNA [39,41,59,60] In order to obtain pure genomic DNA in sufficient quantities for the MethylCap assay, a genomic DNA isolation protocol has been optimized for *Xenopus *embryos (see materials and methods). This protocol yields > 1 μg of genomic DNA from ~100 late blastula/gastrula *Xenopus tropicalis *embryos (Figure 3A). In order to ensure proper resolution of the methylated DNA affinity capture, genomic DNA has to be sonicated to small fragments (200 - 300 bp). *Xenopus *genomic DNA samples were sonicated using a Bioruptor (Diagenode) device and sonicated genomic DNA was saved after five or fifteen minutes of sonication. The samples were then resolved on a 1% agarose gel (Figure 3B). Fifteen minutes of sonication (total volume = 200 μL, concentration = 0,1 μg/μl) yields fragments of 200 - 300 bp. Sonicated genomic DNA was then bound to magnetic beads coated with the MeCP2 MBD domain (Figure 3C) and increasing NaCl concentrations were used to wash and elute the bound methylated DNA fragments. The recovery of methylated DNA was measured by quantitative PCR. The primers used to amplify affinity-precipitated fragments of genomic DNA correspond to DNA methylation-positive (*tcea*, *tfcp2*, *gs17*) and methylation-negative (*Xbra*, *trim33*, *hes4*) loci (Figure 3B). These genomic targets were validated by methylated DNA immunoprecipitation (MeDIP) (Figure 3E). Genomic targets *tcea*, *tfcp2 *and *gs17 *are found positive for DNA methylation by both MeDIP and MethylCap. In line with benchmark studies [40,41] the methylation status of the DNA fragments captured with this technique can be confirmed by bisulfite sequencing [38]. The majority of methylated fragments elute at high molar concentrations of NaCl (500 mM, 700 mM) whereas the non-methylated fragments are washed out at 200 mM, demonstrating the specific methylated-CpG affinity of the MBD resin (Figure 3B). Furthermore, to measure the amount of DNA captured in such an approach, fractions eluted at 500 mM, 600 mM and 700 mM NaCl, were subjected to Picogreen measurement. All the examined fractions had a concentration of > 1 ng/μl (data not shown) making them highly suitable for next generation sequencing library preparation. Taken together, these results demonstrate an efficient approach to study DNA methylation in early vertebrate embryos.

## Discussion

In this study we describe an efficient method for the pull-down of DNA methylation-specific proteins from *Xenopus *oocyte extracts. Furthermore, we demonstrate the functionality of this system by specifically pulling down two major *Xenopus *methyl-CpG binding proteins. In addition, we assay the ability of the methyl-CpG binding domain of MeCP2 to affinity-capture methylated genomic DNA and establish conditions that result in the elution of methylated fractions. Using methylated DNA affinity precipitation, MeCP2 and MBD3 were pulled down from *Xenopus *oocytes and positive as well as negative controls for this approach were established. The chromatin remodelers Mi-2 and ISWI can be used as loading controls and TFIIB as a negative control. Methylated DNA affinity precipitation is a valuable technique that can be coupled to western blotting or HDAC assays in order to assess the composition of bound fractions. Also, a great potential lies in combining methylated DNA pull-downs with mass spectrometry approaches to aid the identification of proteins with DNA methylation-specific binding. A number of recent studies demonstrated the importance of similar applications in identifying novel chromatin components in human cells [30]. The binding activity of methyl-CpG binding proteins in vertebrate oocytes/embryos has not yet been established. For example, in brain extracts, MeCP2 is known to be actively regulated by serine phosphorylation which diminishes its DNA binding affinity and enables the proper neural response [17,19]. It is possible that during early embryogenesis similar phosphorylation events, which result in altered binding affinity, take place. Also, MBD proteins are known to interact with a plethora of proteins and most of these interactions are dependent on the biological context [61]. Methylated DNA affinity precipitation coupled to mass spectrometry might help in identifying the nature of these interactions in the developing embryo. To capture methylated DNA, genomic DNA isolated from early *Xenopus *embryos was incubated with an MBD affinity resin and eluted with increasing salt concentrations. The fractions eluted at concentrations of 500 - 700 mM NaCl contain fragments of highly methylated DNA [38,40,41]. On the other hand, the majority of non-methylated fragments that were non-specifically bound to the resin already elute with 200 mM NaCl. The relatively high concentration of DNA in the fractions renders this method highly suitable for coupling to next generation sequencing technologies.

## Conclusions

Binding of MBDs to sites of genomic DNA methylation is a biologically important process that modulates the transcriptional output of the cell. It is therefore essential to have suitable assays to probe for this kind of interactions. Together, the two affinity-capture based assays discussed above, allow for unbiased profiling of methylated DNA-binding proteins and the genomic loci they may target, setting the stage for a new and exciting era of quantitative profiling in early vertebrate embryos.

## Competing interests

The authors declare that they have no competing interests.

## Authors' contributions

OB and GJCV designed the experiments, and drafted the manuscript. OB Carried out the experiments. GJCV conceived the study and contributed to its design and to writing of the manuscript. Both authors read and approved the final manuscript.

## Statement on animal use

Animal care and use was in accordance with national and European guidelines and standard operating procedures approved by the institutional animal care and use committee (Dierexperimentencommissie, DEC).
